# Review of Neurovascular Reconstruction During Robotic-Assisted Laparoscopic Radical Prostatectomy

**DOI:** 10.1007/s11934-026-01349-w

**Published:** 2026-08-14

**Authors:** Veronica S. Zhang, Shirley L. Wang, Benjamin Schurhamer

**Affiliations:** Division of Urology, University of Pennsylvania Health System, 3400 Civic Center Blvd, Philadelphia, PA 19104 USA

**Keywords:** Robotic-assisted laparoscopic radical prostatectomy, Neurovascular reconstruction, Nerve graft, Neurorrhaphy, Perinatal tissue allografts

## Abstract

**Purpose of Review:**

This manuscript provides a narrative review of techniques of neurovascular reconstruction during robotic-assisted laparoscopic radical prostatectomy (RALP).

**Recent Findings:**

Historic techniques of neurovascular reconstruction, including autologous nerve grafting and neurorrhaphy, have been shown to be feasible in patients in early pilot studies. More recently, perinatal tissue allografts have been studied, with studies supporting earlier recovery of potency or continence.

**Summary:**

We review historic experimental techniques which are less frequently employed, including nerve grafting and end-to-end nerve anastomosis. More recently, perinatal tissue allograft wraps placed over the neurovascular bundles (NVB) have been demonstrated to be feasible, requiring minimal additional operative time. Existing studies demonstrate earlier return to potency or continence for men with good preoperative function. While further prospective research is needed to validate these findings, this represents a feasible method which may accelerate recovery for men undergoing RALP.

## Introduction

Prostate cancer is the second most common malignancy in men, with approximately 300,000 new cases diagnosed each year in the US [1]. Surgical curative treatment for localized disease involves radical prostatectomy. When oncologically feasible, bilateral nerve-sparing radical prostatectomy is the gold standard to allow for faster continence and erectile function. Nerve-sparing techniques include careful intrafascial dissection, avoiding cautery and heat near the NVBs, traction-free dissection to prevent neuropraxia, and real-time pathological screening for positive surgical margins near the NVBs to determine feasibility of NVB preservation [2–5].

Despite advances in nerve-sparing technique, up to 50% of men have extracapsular extension and therefore may require partial or complete resection of the NVB [6, 7]. The most common site of extraprostatic malignancy is the posterolateral portion of the prostate, where the NVBs are located [8, 9]. As a result, the NVBs, which include the cavernous nerves that are responsible for urinary continence and erectile function frequently have to be resected to prevent recurrence of the cancer and positive surgical margins [10]. Urologists must balance a thorough oncologic dissection with preservation of quality of life outcomes.

Prior to implementation of nerve-sparing techniques, nearly all patients undergoing radical prostatectomy faced impotence and up to 50% of patients experienced incontinence 3 months after their radical prostatectomy as opposed to a 25% incontinence rate in bilateral nerve-sparing prostatectomy [10, 11]. With bilateral nerve-sparing RALP, large studies have shown that around 80–90% of men regain potency, with function gradually improving over 1 to 2 years [12, 13]. Even in cases where only one NVB is able to be spared, potency rates are still relatively high at around 69% for previously potent men [14]. Therefore, any nerve-sparing that can be done should be performed. Continence rates are even higher with studies consistently reporting over 95% of men regaining continence and not requiring pads within one year after surgery; there have even been reports of immediate postoperative continence in 86% of men [15–17].

While bilateral nerve-sparing is the preferred method for optimization of functional outcomes, this is not always possible when injury or resection is unavoidable. This review summarizes available reconstructive approaches, including autologous nerve grafting, neurorrhaphy, perinatal tissue allografts, and emerging synthetic biomaterials.

## Autologous Nerve Grafts

The earliest attempt to reconstruct the NVB used sural nerve grafts from the patient’s posterolateral leg to connect the proximal and distal ends of the resected cavernous nerve [18]. Grafts are most frequently used in situations where the two ends of the cavernous nerve are too distant to undergo reanastomosis directly and a longer conduit is needed to promote nerve regeneration and prevent neuropraxia. The harvested sural nerve guides the direction of growth in the proximal regenerating axons until it rejoins with the distal end, and it theoretically should allow for functional return [18].

The sural nerve is most commonly used as the graft since it has an exclusively sensory role, is superficially located and therefore results in minimal surgical morbidity, and its relatively long length allows for ample grafting material without needing to stretch the nerve [19]. However, the genitofemoral and ilioinguinal nerves have also been used to perform autologous grafts. Genitofemoral grafts offer the advantage of being located in the pelvic region and therefore do not require a secondary surgical site to harvest the nerve [20]. But, the genitofemoral nerve is less commonly used as its grafts are usually 5 cm long, which raises concerns over having adequate length for bilateral grafts [21]. On the other hand, the typical length of a sural nerve graft is 10–15 cm, which allows for more grafting potential and decreases the risk of stretch injuries to the nerve [22]. Moreover, the mixed nature of both genitofemoral and ilioinguinal nerves makes it a less popular candidate for grafting than the purely sensory sural nerve.

Despite the initial promise of autologous nerve grafts, the functional outcomes have been modest at best. In the largest study of sural nerve grafting during RALP that included 23 unilateral grafts and 4 bilateral grafts, none of the patients that received bilateral grafts regained potency or returned to their preoperative sexual function [23]. Unilateral grafting resulted in similar functional outcomes to unilateral nerve-sparing, with a little less than half of patients regaining continence and sexual function within 2 years [23]. Several studies have similarly reported minimal functional recovery in bilaterally grafted patients and no functional difference between unilateral sparing or grafting [22, 24, 25].

Due to the limited benefits of nerve grafting, it is neither commonly performed nor recommended. However, much of the work done on nerve preservation and intraoperative handling techniques come from nerve grafting studies, and these techniques are still applied to better preserve nerve function and have inspired more current efforts for neurovascular reconstruction.

## Neurorrhaphy

Neurorrhaphy, the advancement of nerve ends to suture them together, can be performed in cases where the gap between the proximal and distal ends are small, typically < 1 cm [26]. It is critical that the gap is small to allow for a tension-free reconstruction of the nerves. In a case series of 7 patients with high-risk tumors, 5 were able to regain potency sufficient for intercourse at 18 months after neurorrhaphy during a RALP [27]. By only resecting the NVB sections that adhered to the tumor, Martinez-Salamanca et al. were able to preserve enough neuronal material and length to directly reconnect the nerve endings [27]. It is important to note that the remaining NVB was detached from its surrounding tissue in order to shorten the gap between the nerve endings and allow for traction-free suturing [27].

Although there is limited data on direct end-to-end anastomosis in RALPs, pilot studies demonstrate better functional outcomes and recovery compared to using nerve grafts. In a pilot study, Martinez-Salamenca et al. describe recovery to baseline erectile function at 18 months among two patients, three patients with sufficient erectile function with phosphodiesterase-5 inhibitors, and two patients without sufficient erections for intercourse [27]. This could be attributed to there only being one coaptation site in direct neurorrhaphy as opposed to two sites in nerve grafts. Approximately half of regenerating axons are lost when crossing a coaptation site, so substantially fewer axons are regenerating and crossing over to the distal ends of the NVB in nerve grafts [28].

While this technique offers the advantage of not requiring a donor nerve and allows for substantial functional recovery, it is challenging in the context of a radical prostatectomy. Neurorrhaphy and nerve grafting work best for the reconstruction of a single nerve, but the prostatic NVB is more mesh-like and is a fine, microscopic network of nerves that overlies the prostate. Therefore, it is technically challenging to visualize the microscopic nerve endings, and the limited space within the bony pelvis makes the task of delicate and precise suturing extremely difficult. Robotic surgery lends itself especially well to working in tight spaces and deftly maneuvering nerve endings, so direct neurorrhaphy may become more frequently used in RALP cases where most of the NVB can be preserved and there are easily accessible sites of anastomosis.

## Perinatal Tissue Grafts

Traction during surgical intervention leads to tissue scarring and fibrosis around the prostatic region, including the NVB located on the posterolateral prostate [29]. This creates primary damage to the delicate peripheral nerve structures during the procedure, but there is also long-lasting inflammation that occurs in the area during the postoperative period. The inflammatory environment leads to edema, acidosis, and ultimately apoptosis of nerves [29, 30].

By placing dehydrated human amnion/chorion membrane (dHACM) wraps around the spared NVB, inflammatory effects are reduced through the release of cytokines and neurotrophic factors [29, 30]. The membranes also contain growth and repair factors, such as VEGF and FGF that promote the regeneration of axons [30].

Studies have shown that placement of bilateral dHACM wraps in conjunction with bilateral nerve-sparing has been associated with earlier recovery of potency and continence. A comparative longitudinal study with 700 patients undergoing dHACM wraps and 700 with bilateral nerve-sparing but no dHACM showed that potency returned at an average of 35.5 days earlier in the dHACM group (60 ± 61.2 days vs. 95.5 ± 76.2, *p* < 0.05) [31]. Other studies have reported similar shortened times to potency in patients receiving dHACM grafts, with studies showing that the dHACM group on average recovers potency within the first 2 or 3 months after surgery and the non-dHACM group recovers potency after 3 to 4 months [30–32]. Patients with dHACM also had a significantly smaller decline in Sexual Health Inventory for Men (SHIM) scores compared to those with bilateral nerve-sparing but no dHACM. dHACM patients had an average decline of 4.27 points, whereas most patients with radical prostatectomies experience a decline of 9 points or greater [31, 33]. This significantly smaller decrease in SHIM score could explain the faster time to potency as there is less functional deficit to begin with. Razdan et al. reported that dHACM application independently led to a 3.86 higher odds of regaining potency within one year [31]. Younger age (< 55 years old) and dHACM placement resulted in even faster rates of potency recovery [30, 31, 34]. Therefore, younger patients may be especially good candidates for this intervention.

A similar relatively rapid return to continence has been demonstrated with dHACM application. In a retrospective analysis of prospectively collected data, time to continence was 1.2 months in the dHACM group, which was significantly shorter than the 1.8 months in the control group [32]. A longer study conducted at the same institution revealed a median return time to continence of 42 days for patients with dHACM grafts [34].

Additional work in this area has shown that viable cryopreserved umbilical tissue (vCUT) may also be used to improve recovery of continence [35]. A retrospective analysis of patients showed 86% of patients who received vCUT were continent as compared to 74% who underwent standard RALP without vCUT (*p* = 0.04) [35]. This analysis did not note a difference in return of potency. Groups have also experimented with placing placental wraps around the vesicourethral anastomosis in addition to the bilateral NVB [29].

Overall, the results from dHACM studies have shown promise in improving quality of life for patients after radical prostatectomy. It is also a relatively minor addition to the traditional RALP procedure that adds minimal operative time as it requires just two extra steps: cutting the placental graft into two for both NVBs and wrapping it around 90% of the nerve bundle’s circumference [31, 36]. Perinatal tissue allografts have gained increasing interest due to the relative technical feasibility and encouraging functional outcomes, without significant graft-associated adverse effects. Prospective randomized trials evaluating their benefit, however, are limited.

## Synthetic Non-Human Biomaterials

Similar to dHACM membranes, synthetic non-human biomaterials including chitosan membranes can also be applied to periprostatic nerve bundles to improve functional recovery. Chitosan is synthetically derived, providing an alternative to human tissue-derived allografts. In basic science studies, chitosan has been shown to be neuroprotective, anti-inflammatory, hemostatic, antimicrobial, biodegradable, and promote axonal growth of autonomic nerves [37, 38].

Its use in clinical practice is limited as compared to commercially available perinatal tissue products. In a prospective study of 136 patients undergoing nerve-sparing RALP with chitosan grafts and 334 without grafts, there was a significantly faster return of potency during the first two postoperative months in the chitosan group [39]. However, the potency rate was not significantly different after the first two months, implying that chitosan may speed up recovery but not necessarily lead to overall higher potency rates [39]. The grafts can simply be placed over the NVB and left to adhere to the bundle instead of wrapping the membrane around each bundle, so it takes approximately 10 s to apply the chitosan membrane to each NVB [37]. This offers a relatively convenient alternative to dHACM grafts, but additional studies should be done to determine its effects on continence return and long-term potency.

Alternatively, spider silk was used in a 6-person feasibility study as a synthetic nerve graft [40]. Instead of having to be carefully sutured together with the proximal and distal ends of the NVB, the spider silk graft was just placed over the nerve endings and allowed to serve as a biodegradable, guiding path for Schwann cell regeneration [40, 41]. This cuts down on operative time and also should lead to better results as there is no further nerve injury from the suturing process. After 18 months, 3 out of the 6 men regained adequate erections for intercouse, and their SHIM scores were only 1.5 points below their preoperative baseline [40]. Spider silk grafts offer a unique solution to the issue of grafting two nerves end-to-end. Since the NVB is described as a diffuse plexus of nerves rather than a single nerve, it may be best to use a material that can be laid over the general area to guide axonal regrowth instead of the prior sural nerve grafts that required precise reconnections but yielded modest results. Ultimately, larger studies are needed to fully evaluate the clinical role of spider silk and other synthetic, pro-regeneration materials in prostatectomies.

## Conclusion

It is critical to balance the oncologic and functional outcomes for men undergoing surgical treatment of prostate cancer. While bilateral nerve preservation remains the gold standard for optimizing functional outcomes, this is not always oncologically feasible. A range of strategies to reconstruct or augment spared NVBs have been explored: autologous nerve grafts, neurorrhaphy, human-derived placental wraps, and synthetic materials that promote healing and regeneration. Currently, perinatal tissue and synthetic grafts appear to aid in recovery of erectile function or continence without significant added technical complexity. Larger prospective comparative studies with long-term follow-up are needed to evaluate the true benefit of these grafts.

## Data Availability

No datasets were generated or analysed during the current study.
